# The Common Task

**Published:** 1908-04-18

**Authors:** 


### Nursing Administration.

### THE COMMON TASK.

### Progress in Canada.

It causes a movement of surprise to learn that the oldest training school in Canada is that at the Toronto General Hospital, and that the date of its founding is 1881. All the marvellous developments of Canadian nursing have taken place in the compass of twenty-seven years. True, a good deal of progress has taken place in this country since 1881, but from the point of view of solid organisation and *esprit de corps* we are still centuries behind our daughters over the water. Much may be accounted for by the absence of bad traditions in the new country, but still more must be laid to the account of the undaunted enthusiasm and fresh vitality of the Canadian women. We are sometimes reduced to wishing that some of the wholesome breezes which have inspired such excellent work could stir the pulses of those conventional probationers in English hospitals whose aspirations are conspicuous by their absence.

### Hygiene as Preliminary Training.

The syllabus of examinations to be held this year in June and December by the Incorporated Institute of Hygiene, 34 Devonshire Street, W., presents many features of special interest to those who desire to become nurses. The examinations are intended for women only, and candidates for the diploma of the institute must be over eighteen years of age. The subjects selected for study are seven in number—namely, the hygiene of the home, the hygiene of motherhood, the feeding and rearing of children, food and cooking, home nursing and first aid, school hygiene, and physical training. No lectures are given in connection with the examinations, but text-books are recommended to students, and they are at liberty to attend such courses of instruction as they may choose. It is common to most matrons to be besieged by applications from girls who are looking forward eventually to being nurses, and who desire to be advancing their aim, although far too young to begin training. These examinations might usefully aid to bridge the gulf between school and hospital for many probationers.

### Too Many Lectures.

Complaints are heard in America of over-enthusiasm on the part of the medical staff in certain hospitals, which takes the form of administering incessant lectures to the nurses on medical and surgical subjects. The result is what an experienced matron stigmatises as "random training." In one hospital it is stated that each of the eleven members of the medical staff offered a course of lectures to the pupil nurses, and that each offer was accepted by the authorities for fear of giving offence. We can cordially agree that it would have benefited the poor probationers more to be playing tennis than to be listening to elaborate discourses on the structure of the ear and the obscure diseases to which it is exposed. This kind of over-head teaching is familiar enough in some hospitals on this side of the water, and proceeds from an inability on the part of the lecturer to distinguish between the things nurses want to know and the things which are purely of medical interest. The most essential part of the nurse's curriculum lies in the knowledge of what to omit.

### Mental and Sick Nursing.

A text-book by Dr. Robert Jones, the resident physician and superintendent of Claybury Asylum, on "Mental and Sick Nursing" (Scientific Press, 3s. 6d.) will be welcomed as an invaluable aid in asylums all over the country. It is indubitably the best work of the kind yet issued to the public. For Dr. Jones has handled his subject as physician and as superintendent, that is to say, he has dealt with the duties both separate and combined of the sick nurse, the mental nurse, and the asylum attendant. These three sets of duties, frequently combined in one person, have never before been so clearly distinguished, nor so admirably detailed. Incidentally, the volume furnishes data which cannot fail to be of the utmost value to institutions as yet unprovided with the excellent system of asylum management presented at Claybury. The introductory chapters on physiology, and especially those on brain processes, present an immense deal of most valuable matter in a highly condensed and clear manner. They are, we think, likely to be of the utmost service as a basis for lectures to nurses, and several admirable courses could be framed with their assistance. The remainder of the volume is taken up with the duties of the mental nurse, in asylums and in private houses. There is a chapter on the care of the epileptic and defective, and there are many well-thought-out pages on the treatment of various forms of physical disease in the insane. Diseases of the respiratory organs, diseases of the abdominal organs, fevers, skin affections, poisoning, fractures, dislocations, and hæmorrhage are carefully considered from the point of view of the mental nurse, who will find no surer guide in the performance of her duties. The directions are the outcome of long personal experience and experiment in the interests of the mentally afflicted, and mark an immense advance in rational and humane treatment. Dr. Jones is a great advocate for the use of physical drill in asylums, and considers that it should not be neglected among private patients. His concluding words will be read with appreciation by all those who are engaged in this arduous and highly responsible work:—

"The demand for mental nurses is great and steadily increasing. Into whatever sphere of life a sympathising and well-trained nurse enters, there the standard of life is raised, for she brings enlightenment upon such subjects as cleanliness, self-control, thrift, and the care of the young. If her own standard is high she becomes the most convincing health missioner that can influence any community, and she can, more than any other agent, help to 'render growth more perfect, decay less rapid, life more vigorous, and death more remote.'"

